# Preparative Separation of Monoterpenoid Indole Alkaloid Epimers from *Ervatamia yunnanensis* Tsiang by pH-Zone-Refining Counter-Current Chromatography Combined with Preparative High-Performance Liquid Chromatography

**DOI:** 10.3390/molecules24071316

**Published:** 2019-04-03

**Authors:** Jie Zhou, Si-Yu Du, Hong-Jing Dong, Lei Fang, Jin-Hong Feng

**Affiliations:** 1 School of Biological Science and Technology, University of Jinan, Jinan 250022, China; zhoujie8761@163.com; 2 Key laboratory of Natural Pharmaceutical Chemistry, Shandong University of Traditional Chinese Medicine, Jinan 250200, China; dsy_fine@163.com; 3 Shandong Key Laboratory of TCM Quality Control Technology, Shandong Analysis and Test Center, Qilu University of Technology (Shandong Academy of Sciences), Jinan 250014, China; donghongjing_2006@163.com

**Keywords:** *Ervatamia yunnanensis*, monoterpenoid indole alkaloids, epimers, pH-zone-refining counter-current chromatography, high-performance liquid chromatography

## Abstract

An effective method was developed for the preparative separation and purification of monoterpenoid indole alkaloid epimers from *Ervatamia yunnanensis* Tsiang using a combination of pH-zone-refining counter-current chromatography and preparative high-performance liquid chromatography. With this method, two pairs of MIA epimers including ervatamine (72 mg, **1**), 20-*epi*-ervatamine (27 mg, **4**), dregamine (95 mg, **2**), tabernaemontanine (129 mg, **3**), along with two MIAs, apparicine (112 mg, **5**) and isovoacangine (15 mg, **6**), were successfully purified from 2.1 g crude extract of *E. yunnanensis*, each with a purity of over 95% as determined by HPLC. The structures of the MIAs were identified by ESI-MS, 1D, and 2D NMR.

## 1. Introduction

Monoterpenoid indole alkaloids (MIAs) are an important group of alkaloids in plants that are mainly elaborated by the plants of the *Apocynaceae*, *Rubiaceae*, and *Loganiaceae* families [1]. These alkaloids originating from the condensation of tryptophan with secologanin have attracted significant interests of synthetic organic chemists due to their structural diversity and complicated biosynthesis [2,3,4,5]. Many of these alkaloids are also outstanding for their pharmacological significance, such as reserpine, camptothecin, and vincristine [6]. Plants of the genus *Ervatamia* (*Apocynaceae*), which comprise about 120 species, are regarded as a good source of MIAs [7]. To date, more than 431 MIAs have been isolated from the genus and have shown significant cytotoxic, anticholinesterase, and antiplasmodial activities [8,9,10]. Due to the existence of multiple chiral carbon atoms in the structure, these MIAs often have many epimers [11]. However, the conventional methods for the separation of MIA epimers were often based on repeated column chromatography, which always led to poor separation effect, low recovery, and a requirement of high quantities of solvents. Therefore, it is necessary to develop an effective method to separate and purify large quantities of MIA epimers with high purity for further pharmacological research.

**Figure 1:**
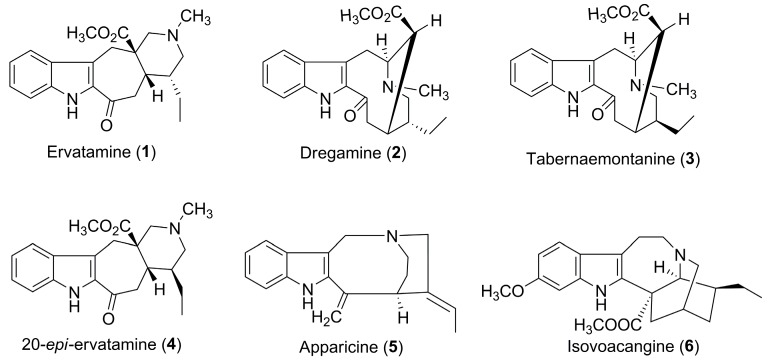
Chemical structures of monoterpenoid indole alkaloids (MIAs) from *E. yunnanensis*.

pH-zone-refining counter-current chromatography (pH-ZRCCC), as a large-scale preparative technique, is a modified high-speed counter-current chromatography that allows for the separation of basic or acidic compounds into a succession of highly concentrated rectangular peaks fused together with minimum overlapping impurities [12]. This method presents great advantages over conventional column chromatography, such as no irreversible adsorption, large load capacity, excellent sample recovery, minimum overlap of rectangular peaks, and concentrated impurities at rectangular peaks boundaries, and has been widely used for the preparative separation and purification of alkaloids and organic acids from medicinal plants [13,14]. Occasionally, pH-ZRCCC could not provide a satisfying resolution due to the structural similarity of target compounds. Preparative high-performance liquid chromatography (prep-HPLC), a classical chromatographic method, has been extensively applied in the separation and purification of natural products. To the best of our knowledge, there were few reports on the preparative separation of MIA epimers from plant extracts using a combination of pH-ZRCCC and prep-HPLC. In the present paper, an efficient method using pH-ZRCCC combined with prep-HPLC was developed for the preparative separation of two pairs of MIA epimers composed of ervatamine (**1**), 20-*epi*-ervatamine (**4**), dregamine (**2**), and tabernaemontanine (**3**), together with two MIAs, apparicine (**5**) and isovoacangine (**6**), from *E. yunnanensis* Tsiang for the first time (Figure 1).

## 2. Results

### 2.1. Selection of the Two-Phase Solvent Systems

The selection of the two-phase solvent system is the first and critical step in pH-ZRCCC separation. A suitable solvent system should provide an ideal partition coefficient and good sample solution. The *K*_base_ (*K*-value in the basified solvent system) should be much bigger than 1.0 and the *K*_acid_ (*K*-value in the acidized solvent system) much smaller than 1.0. The methyl tert–butyl ether–acetonitrile–water (MAWat) is the most frequently cited solvent system in pH-ZRCCC separation [12]. However, the crude sample of *E. yunnanensis* could not properly dissolve in the MAWat solvent system. Hexane–ethyl acetate–methanol–water (HEMWat), as an alternative solvent system for pH-ZRCCC separation of alkaloids from plant extracts, would provide good solubility of the sample. Thus, HEMWat solvent systems at different solvent ratios (3:7:1:9, 5:5:2:8, *v*/*v*) were evaluated in our research and provided good sample solution and suitable *K*_base_ and *K*_acid_ values, which could be suitable for pH-ZRCCC separation.

### 2.2. Preliminary Separation by pH-ZRCCC

**Figure 2:**
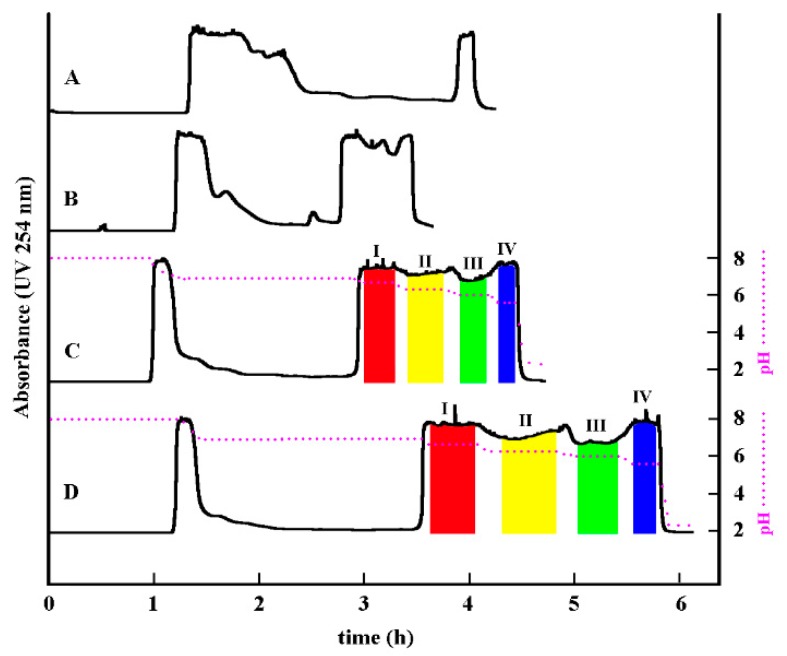
pH-zone-refining counter-current chromatography (pH-ZRCCC) chromatogram of the crude sample of *E. yunnanensis*. (**a**) hexane–ethyl acetate–methanol–water (3:7:1:9, *v/v*), 0.7g of sample, 10 mM triethylamine (TEA) in upper phase, and 10 mM HCl in lower phase; (**b**) hexane–ethyl acetate–methanol–water (5:5:2:8, *v/v*), 0.7g of sample, 10 mM TEA in upper phase, and 10 mM HCl in lower phase; (**c**) hexane–ethyl acetate–methanol–water (5:5:2:8, *v/v*), 0.8g of sample, 20 mM TEA in upper phase, and 5 mM HCl in lower phase; (**d**) hexane–ethyl acetate–methanol–water (5:5:2:8, *v/v*), 2.1 g of sample, 20 mM TEA in upper phase, and 5 mM HCl in lower phase. Experimental conditions: revolution speed: 850 r/min; flow rate: 2.0 mL/min; UV detection wavelength: 254 nm.

The crude sample (0.7g) was initially separated by pH-ZRCCC using hexane–ethyl acetate–methanol–water (3:7:1:9, *v*/*v*) with 10 mM triethylamine (TEA) in upper organic phase and 10 mM HCl in lower aqueous phase (Figure 2a), which showed a certain characteristic pattern of pH-ZRCCC. However, the MIAs were eluted too fast with poor resolution. Then, the solvent system hexane–ethyl acetate–methanol–water (5:5:2:8, *v*/*v*) was evaluated, which provided better resolution (Figure 2b). Considering the observed elution results in Figure 2b, the resolution would be improved by increasing the amount of TEA in upper organic phase and decreasing the amount of HCl in lower aqueous phase. Thus, hexane–ethyl acetate–methanol–water (5:5:2:8, *v*/*v*) with 20 mM TEA in upper organic phase and 5 mM HCl in lower aqueous phase was tested, which showed a typical pH-ZRCCC for the separation of the crude alkaloids (Figure 2c). As shown in Figure 2c, the target MIAs were eluted as irregular rectangular peaks combined with impurities or minor components highly concentrated at their boundaries, suggesting the successful pH-ZRCCC separation of the sample. Then, 2.1 g of crude sample was separated by pH-ZRCCC separation using the solvent system hexane–ethyl acetate–methanol–water (5:5:2:8, *v*/*v*) (Figure 2d). The concentration of TEA was 20 mM in upper organic phase and HCl was 5 mM in lower aqueous phase. As a result, two MIAs, 112 mg of apparicine (fraction I, **5**) and 129 mg of tabernaemontanine (fraction III, **3**), together with the partially purified fraction II (**1** and **2**) and fraction IV (**4** and **6**).

### 2.3. Subsequent Purification by Prep-HPLC

**Figure 3:**
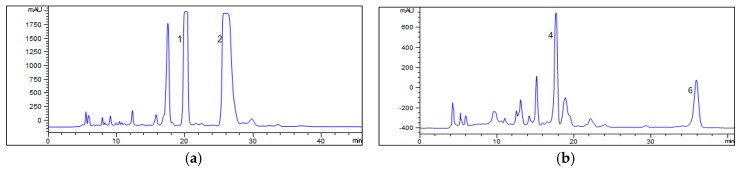
Preparative high-performance liquid chromatography (Prep-HPLC) chromatograms of fraction II (**a**) and fraction VI (**b**). Conditions: column, YMC-Triart C_18_ (250 × 9.6 mm, i.d., 5 μ); flow rate, 2.0 mL/min; UV wavelength, 254 nm; mobile phase for fraction II, methanol-0.3% aqueous acetic acid (75:25, *v*/*v*); mobile phase for fraction VI, acetonitrile-0.3% aqueous acetic acid (75:25, *v*/*v*).

The partially purified fractions II (348 mg) and VI (153 mg) were further purified by prep-HPLC, respectively, which led to the separation of 72 mg of ervatamine (**1**), 95 mg of dregamine (**2**), 27 mg of 20-*epi*-ervatamine (**4**), and 15 mg of isovoacangine (**6**) (Figure 3).

### 2.4. HPLC Analysis and Structural Identification of Purified MIAs

**Figure 4:**
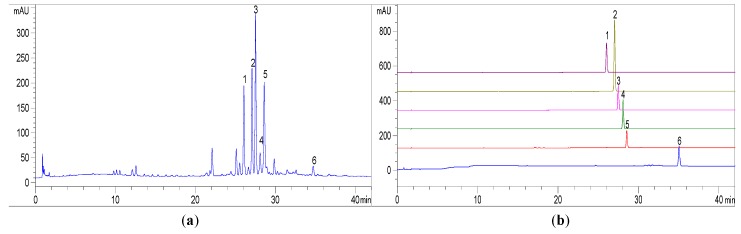
(**a**) HPLC analysis of the crude sample of *E. yunnanensis*. (**a**); (**b**) HPLC chromatograms spectra of MIAs (**1**–**6**). Conditions: YMC-Triart C_18_ (150 × 9.6 mm, i.d., 5 μ); flow rate, 1.0 mL/min; UV wavelength, 254 nm; solvent A (acetonitrile) and B (0.3% *v*/*v* aqueous acetic acid) in gradient elution as follows: 0–8 min, 42% A; 8–32 min, 42% A–50% A; 32–35 min, 50% A.

The purities of the two pairs of MIA epimers, ervatamine (**1**), 20-*epi*-ervatamine (**4**), dregamine (**2**), and tabernaemontanine (**3**), together with apparicine (**5**) and isovoacangine (**6**), were determined as 99.2%, 99.1%, 98.7%, 98.9%, 99.1%, and 98.2% by HPLC analysis, respectively (Figure 4).

The structures of the purified MIAs were identified by comparison of their ESI-MS, 1D, and 2D NMR data with those in the literature.

Compound **1**: ESI-MS *m*/*z*: 355 [M + H]^+^. ^1^H and ^13^C NMR data (see Table 1). The planar structure of **1** was established as shown by comparison of their ESI-MS, ^1^H, and ^13^C NMR data with those in the literature [15]. Furthermore, the ROESY correlations of H-14/H-20, H-14/H-19, and H-6/H-15 confirmed that **1** possessed the same relative configurations at C-15, C-16, and C-20 as those of ervatamine.

Compound **2**: ESI-MS *m*/*z*: 355 [M + H]^+^. ^1^H and ^13^C NMR data (see Table 1). The ^1^H and ^13^C NMR data of **2** were in accordance with literature values of dregamine [16]. H-21b was observed as a triplet (*J* = 12.3 Hz) in the ^1^H NMR spectrum, which suggested that the H-20 was β-oriented [11]. The ROESY correlations of H-14/H-20, and H-15/H-18 indicated that **2** possessed the same relative configurations as those of dregamine.

Compound **3**: ESI-MS *m*/*z*: 355 [M + H]^+^. ^1^H and ^13^C NMR data (see Table 2). The ^1^H and ^13^C NMR data suggested **3** was a diastereisomer of **2**. The ^1^H NMR spectrum showed a doublet of doublets at *δ*_H_ 3.19 (*J* = 12.9, 3.8 Hz, H-21b), suggesting that the H-20 was *α*-oriented in **3**. Consequently, **3** was determined to be tabernaemontanine [17].

Compound **4**: ESI-MS *m*/*z*: 355 [M + H]^+^. ^1^H and ^13^C NMR data (see Table 2). When its ^1^H and ^13^C NMR data were compared with those of **1**, small differences in the chemical shifts of C-14, C-15, and C-20 were observed. Furthermore, the ROESY correlations of H-14/H-20 and H-6/H-15 suggested the relative configurations at C-15, C-16, and C-20 in **4** were the same as those in 20-*epi*-ervatamine [18]. Accordingly, compound **4** was an epimer of **1**.

Compound **5**: ESI-MS *m*/*z*: 265 [M + H]^+^. ^1^H NMR (CDCl_3_, 600 MHz) *δ*: 7.85 (1H, s, H-1), 7.42 (1H, d, *J* = 8.0 Hz, H-9), 7.23 (1H, d, *J* = 8.0 Hz, H-12), 7.18 (1H, t, *J* = 8.0 Hz, H-11), 7.06 (1H, t, *J* = 8.0 Hz, H-10), 5.39 (1H, s, H-17b), 5.26 (1H, s, H-17a), 5.24 (1H, q, *J* = 6.6 Hz, H-19), 4.50 (1H, d, *J* = 17.6 Hz, H-6b), 4.26 (1H, d, *J* = 17.6 Hz, H-6a), 3.91 (1H, m, H-15), 3.81 (1H, dd, *J* = 15.9, 2.4 Hz, H-21b), 3.41 (1H, m, H-3b), 3.20 (1H, d, *J* = 15.9 Hz, H-21a), 3.06 (1H, m, H-3a), 2.16 (1H, m, H-14b), 1.90 (1H, m, H-14a), 1.46 (3H, d, *J* = 6.6 Hz, H-18). ^13^C NMR (CDCl_3_, 150 MHz) *δ*: 12.6 (C-18), 29.7 (C-14), 41.3 (C-15), 45.3 (C-3), 54.3 (C-6 and C-21), 110.2 (C-12), 111.4 (C-7), 112.2 (C-17), 118.6 (C-9), 119.3 (C-10), 122.9 (C-11), 128.9 (C-8), 135.6 (C-13), 145.2 (C-2). The ^1^H and ^13^C NMR data of **5** was in agreement with those of apparicine [19].

Compound **6**: ESI-MS *m*/*z*: 369 [M + H]^+^. ^1^H NMR (CDCl_3_, 600 MHz) *δ*: 7.13 (1H, d, *J* = 8.7 Hz, H-9), 6.90 (1H, d, *J* = 2.4 Hz, H-12), 6.69 (1H, dd, *J* = 8.7, 2.4 Hz, H-10), 3.80 (3H, s, OCH_3_), 3.69 (3H, s, COOCH_3_), 3.59 (1H, brs, H-21), 3.38 (1H, m Hz, H-5b), 3.07 (1H, m, H-5a), 3.06 (1H, m, H-6b), 2.99 (1H, m, H-6a), 2.92 (1H, m, H-3b), 2.78 (1H, dt, *J* = 8.5, 1.8 Hz, H-12), 2.70 (dt, *J* = 13.5, 2.2 Hz, H-17b), 1.92 (1H, m, H-17a), 1.84 (1H, m, H-14), 1.76 (1H, m, H-15b), 1.56 (1H, m, H-19b), 1.44 (1H, m, H-19a), 1.37 (1H, m, H-20), 1.13 (1H, m, H-5a), 0.90 (3H, d, *J* = 7.4 Hz, H-18). ^13^C NMR (CDCl_3_, 150 MHz) *δ*: 12.1 (C-18), 22.8 (C-6), 27.9 (C-19), 28.9 (C-14), 33.2 (C-15), 37.1 (C-17), 40.1 (C-20), 52.9 (COOCH_3_), 54.2 (C-3), 54.8 (C-5), 56.3 (OCH_3_), 56.4 (C-16), 57.9 (C-21), 101.1 (C-12), 110.6 (C-13), 112.2 (C-9), 112.3 (C-10), 129.9 (C-7), 132.8 (C-8), 139.3 (C-2), 154.9 (C-11), 176.3 (COOCH_3_). The ^1^H and ^13^C NMR data of **6** was in agreement with those of isovoacangine [20].

^1^H and ^13^C-NMR (Figures S1–S18) spectra of these compounds are available in the Supplementary Materials.

## 3. Materials and Methods

### 3.1. Apparatus

The pH-ZRCCC was carried out using a TBE-300C high-speed countercurrent chromatography apparatus (Tauto Biotech, Shanghai, China) with a series of three multilayer coils columns (total capacity = 300 mL) and a 20 mL sample loop. The above system was also equipped with a TBP-5002S constant-flow pump (Tauto Biotech, Shanghai, China), a TBD2000 UV detector (Tauto Biotech, Shanghai, China), a HW2000 workstation (Tauto Biotech, Shanghai, China), and a DC-0506 circulatory temperature regulator (Tauto Biotech, Shanghai, China). HPLC analyses and separations were performed on an Agilent 1260 infinity quaternary LC instrument (Agilent Technologies, Waldbronn, Germany) using a YMC-Triart (4.6 × 150 mm) column and a YMC-Triart C_18_ (9.6 × 250 mm) column. ESI-MS analyses were performed using an Agilent 1100/MSG1946 (Agilent, Santa Clara, CA, USA). The 1D and 2D NMR spectra were recorded on a Bruker AVANCE DRX600 NMR spectrometer (Bruker BioSpin Corporation, Fällanden, Switzerland) with trimethylsilane (TMS) as the internal standard.

### 3.2. Reagents and Materials

Organic solvents used for sample preparation and pH-ZRCCC separation were of analytical grade and purchased from Tianjing Chemical Factory (Tianjing, China). Methanol used for analytical and preparative HPLC was of chromatographic grade and purchased from Siyou Special Reagent Factory (Tianjin, China). Deionized water was prepared by a Milli-Q system (Millipore, Bedford, MA, USA). Normal phase column chromatography was performed on silica gel (200–300 mesh, Qingdao Marine Chemical Inc., Qingdao, China).

The stems of *E. yunnanensis* Tsiang were collected from Puer city of Yunnan, China and identified by Prof. Jia Li (College of Pharmacy, Shandong University of Traditional Chinese Medicine, Jinan, China).

### 3.3. Preparation of Crude Sample

The powdered stems of *E. yunnanensis* Tsiang (5.0 kg) were extracted with 95% EtOH (3 × 50 L) at room temperature. After filtration, the extract was combined and evaporated in vacuo to afford a crude extract (210 g). The crude extract was partitioned between 2% HCl solution and EtOAc. The acidic water-soluble portion was adjusted to pH 10 by a 10% ammonia solution and then extracted with CHCl_3_ to give an alkaloidal extract (72 g). The alkaloidal extract was subjected to a silica gel column eluted with a gradient of CH_2_Cl_2_-MeOH (from 200:1 to 10:1) to afford seven fractions, A–G. Fraction C (4.2 g) was evaporated to dryness and used for subsequent pH-ZRCCC separation.

### 3.4. Selection and Preparation of Two-Phase Solvent Systems

The selection of an optimal two-phase solvent system is the critical step for a successful pH-ZRCCC separation. According to the golden rules introduced by Ito, the optimal solvent system should provide ideal values of *K*, which need to meet *K*_base_ >> 1 and *K*_acid_ << 1 [12]. In the study, the *K*_base_ and *K*_acid_ was observed from TLC plates. The solvent system of hexane–ethyl acetate–methanol–water (5:5:2:8, *v*/*v*) was finally selected for the pH-ZRCCC separation. The solvent system of hexane–ethyl acetate–methanol–water (5:5:2:8, *v/v*) was equilibrated in a separated funnel and separated into two phases. The upper phase was used as the stationary phase by adding 20 mM TEA, while the lower phase was used as the mobile phase by adding acidified with 10 mM HCl. The sample solution was prepared by dissolving the fraction C (2.1 g) in the 15 mL of the basic upper phase and 15 mL of the lower phase without HCl.

### 3.5. Preliminary Separation by pH-ZRCCC

The multilayer coiled column of TBE-300C HSCCC apparatus was filled entirely with the upper organic phase as the stationary phase and rotated at 850 rpm. The lower aqueous phase was then pumped into the column at a suitable flow-rate of 2 mL/min in head-to-end mode. The sample was injected into the column through the inlet when hydrodynamic equilibrium was reached. The effluent from the outlet of the column was continuously monitored by a UV detector at 254 nm and manually collected at 5 min intervals. After the separation was completed, the pH values of each fraction were measured by a pH meter. Finally, the solvent in the column was pushed into a measuring cylinder with pressurized nitrogen gas. The retention of the stationary phase was measured as the volume of the residual stationary phase (197 mL) divided by the column volume.

### 3.6. Subsequent Separation by Prep-HPLC

The partially purified fractions II and IV from pH-ZRCCC separation were further purified by prep-HPLC on a YMC-Triart C_18_ (9.6 × 250 mm) column at room temperature. The flow rate was set at 2.0 mL/min, and the effluent was monitored at 254 nm. For fraction II, the mobile phase was methanol-0.3% aqueous acetic acid (75:25, *v*/*v*), while the mobile phase was acetonitrile-0.3% aqueous acetic acid (75:25, *v*/*v*) for fraction VI.

### 3.7. HPLC Analysis of Samples, pH-ZRCCC, and Prep-HPLC Fractions

The samples and each fraction from pH-ZRCCC and prep-HPLC separation were analyzed by HPLC using a YMC-Triart C_18_ (4.6 × 150 mm) column at a flow-rate of 1.0 mL/min. The mobile phase was methanol (solvent A) and 0.1% acetic acid (solvent B) with the following gradient: 0 min 40% A; 45 min 75% A. The detection wavelength was 254 nm.

### 3.8. Identification of Isolated MIAs

The identification of isolated MIAs was determined by MS, 1D, and 2D NMR. ESI-MS spectra were carried out on an Agilent 1100/MS-G1946 (Agilent, Santa Clara, CA, USA) mass spectrometer in the negative ionization mode. The 1D and 2D NMR spectra were measured on a Bruker AVANCE DRX600 NMR spectrometer (Bruker BioSpin Corporation, Fällanden, Switzerland).

## 4. Conclusions

In the present study, the preparative separation and purification of MIA epimers from *E. yunnanensis* by using a combination of pH-ZRCCC and prep-HPLC was investigated. The MIAs were initially enriched and separated from the crude sample by pH-ZRCCC, followed by further purification by prep-HPLC. As a result, two pairs of MIA epimers including ervatamine, 20-*epi*-ervatamine, dregamine, and tabernaemontanine, along with two MIAs, apparicine and isovoacangine, were successfully purified with high-purity from 2.1 g crude extract. The most significant advantage of this method was that good separation effects and larger load capacity of MIAs were achieved in a short time. The results suggested that the combination of pH-ZRCCC with prep-HPLC is a suitable and effective protocol for separation of MIA epimers from extremely complex herbs.

## Figures and Tables

**Table 1:** ^1^H and ^13^C NMR data of compounds **1** and **4** (in CDCl_3_).

Position	1			4	
	*δ*_C_	*δ*_H_ (*J* in Hz)		*δ*_C_	*δ*_H_ (*J* in Hz)
1		8.84 s			8.71 s
2	132.7			132.4	
3	194.0			192.5	
5a	60.7	2.08 d (11.7)		65.1	2.42 d (11.0)
5b		3.46 d (11.7)			2.81 dd (11.0, 1.5)
6a	31.7	2.81 d (15.8)		29.3	3.67 d (17.7)
6b		3.49 d (15.8)			3.75 d (17.7)
7	119.4			122.3	
8	127.4			128.3	
9	120.1	7.55 d (8.2)		120.1	7.71 d (8.0)
10	120.6	7k.13 t (8.2)		121.4	7.13 t (8.0)
11	126.5	7.33 t (8.2)		126.6	7.33 t (8.0)
12	112.2	7.39 d (8.2)		111.8	7.34 d (8.0)
13	136.5			136.5	
14a	36.6	2.49 dd (15.5, 10.5)		43.2	2.89 d (5.2)
14b		2.60 d (15.5)			
15	36.2	2.57 dd (10.5, 4.5)		41.0	2.23 dd (10.5, 5.2)
16	49.3			51.0	
18	11.4	0.88 t (7.4)		10.7	0.86 t (7.4)
19a	23.9	1.37 m		23.8	1.63 m
19b					1.21 m
20	39.1	1.85 m		37.8	1.72 m
21a	57.5	1.57 d (10.9)		60.4	1.78 d (10.9)
21b		2.64 dd (10.9, 3.8)			2.86 dd (10.9, 3.7)
N-CH_3_	46.3	2.31 s		46.3	2.32 s
CO*OCH*_3_	52.4	3.51 s		52.1	3.51 s
*CO*OCH_3_	175.5			175.7	

**Table 2:** ^1^H and ^13^C NMR data of compounds **1** and **4** (in CDCl_3_).

Position	2			3	
	*δ*_C_	*δ*_H_ (*J* in Hz)		*δ*_C_	*δ*_H_ (*J* in Hz)
1		8.85 s			8.89 s
2	134.0			133.9	
3	191.5			190.8	
5	56.7	3.97 m		56.7	2.70 m
6a	20.1	3.24 d (14.7, 10.0)		18.5	3.30 dd (14.6, 8.2)
6b		3.38 dd (14.7, 8.2)			3.46 dd (14.6, 9.8)
7	120.4			120.7	
8	128.5			128.6	
9	120.9	7.70 d (8.0)		120.9	7.70 d (8.2)
10	120.3	7.15 t (8.0)		120.3	7.14 t (8.2)
11	111.7	7.33 m (8.0)		111.7	7.32 m
12	126.7	7.33 m (8.0)		126.6	7.32 m
13	136.3			136.3	
14a	39.3	2.16 dd (13.0, 6.9)		45.7	2.75 d (12.2, 6.9)
14b		3.12 d (13.0)			3.43 d (12.2)
15	30.6	2.89 m		31.8	2.23 dd (10.5, 5.2)
16	49.1	2.59 dd (12.7, 4.3)		43.5	3.01 t (3.3)
18	11.4	1.01 t (7.4)		12.8	0.97 t (7.2)
19a	23.5	1.36 m		25.4	1.52 m
19b					1.72 m
20	43.5	1.89 m		42.6	1.51 m
21a	48.8	2.59 d (12.3, 4.3)		46.6	2.48 d (12.9)
21b		2.78 t (12.3)			3.19 dd (12.9, 3.8)
N-CH_3_	42.6	2.62 s		43.1	2.56 s
CO*OCH*_3_	50.4	2.64 s		50.3	2.61 s
*CO*OCH_3_	171.4			172.1	

## Supplementary Materials

The supplementary materials are available online.
