# Supplementary material for: Preparative Separation of Monoterpenoid Indole Alkaloid Epimers from Ervatamia yunnanensis Tsiang by pH-Zone-Refining Counter-Current Chromatography Combined with Preparative High-Performance Liquid Chromatography
Source: Molecules. 2019 Apr 3;24(7):1316. doi: 10.3390/molecules24071316 (PMC6480700; doi:10.3390/molecules24071316)

Supplementary material for

Preparative separation of monoterpenoid indole alkaloid epimers from *Ervatamia yunnanensis* Tsiang by pH-zone-refining counter-current chromatography combined with preparative high-performance liquid chromatography

Jie Zhou [a,1], Si-Yu Du [b,1], Hong-Jing Dong [c], Lei Fang [a, b,*], Jin-Hong Feng [c,*]

[a] School of Biological Science and Technology, University of Jinan, Jinan 250022, China; zhoujie8761@163.com (J.Z.)

[b] Key laboratory of Natural Pharmaceutical Chemistry, Shandong University of Traditional Chinese Medicine, Jinan, 250200, China; dsy_fine@163.com (S.D.)

[c] Shandong Key Laboratory of TCM Quality Control Technology, Shandong Analysis and Test Center, Qilu University of Technology (Shandong Academy of Sciences), Jinan 250014, China; donghongjing_2006@163.com (H.D.)

* Correspondence: fleiv@163.com (L.F.), Tel: 0086-531-89736799; fengjinhong520@163.com (J.F.), Tel: 0086-531- 68606193

[1] J. Zhou and S.Y. Du contributed equally to this article.

Figure S1. ESIMS of compound **1**

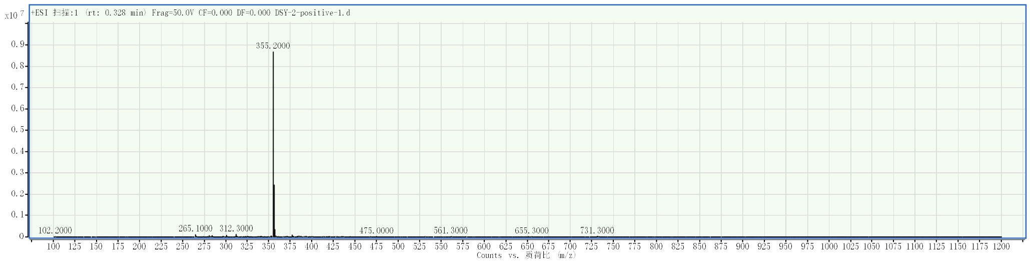

Figure S2. ^{1}H NMR spectrum of compound **1** in $CDCl_3$ (600 MHz)

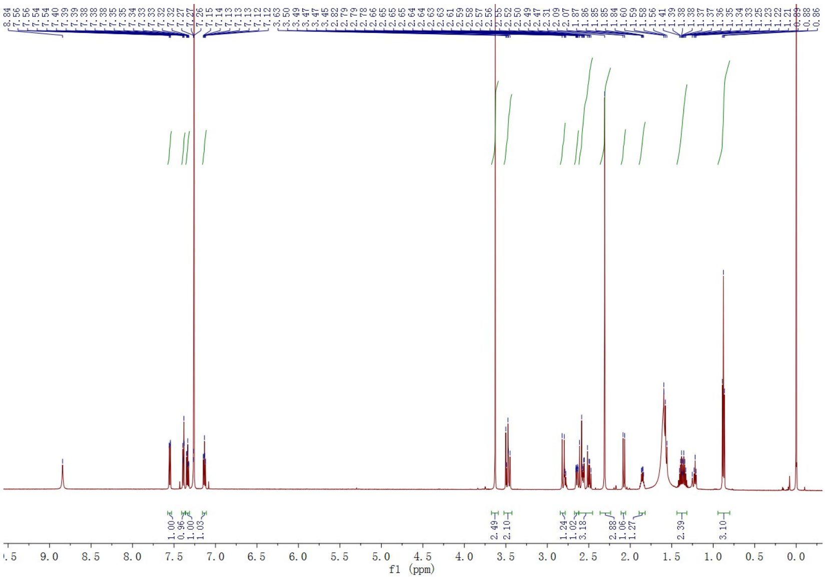

Figure S3. ^{13}C NMR spectrum of compound **1** in $CDCl_3$ (150 MHz)

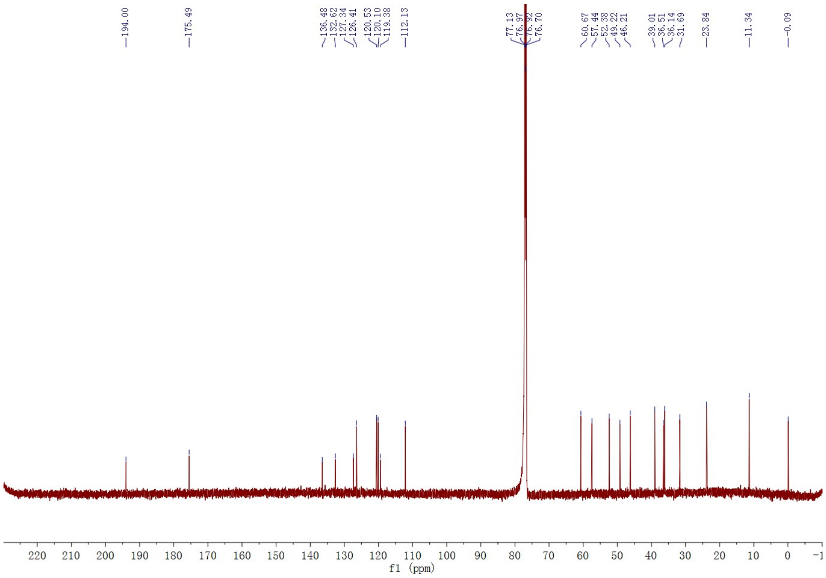

Figure S4. ESIMS of compound **2**

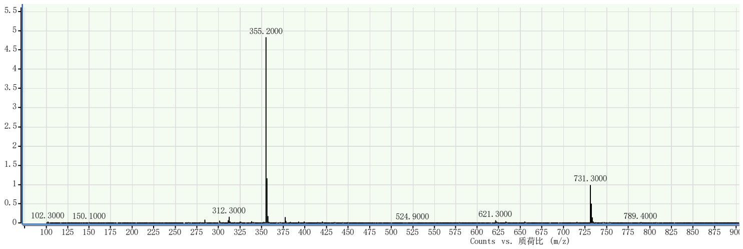

Figure S5. ^{1}H NMR spectrum of compound **2** in $CDCl_3$ (600 MHz)

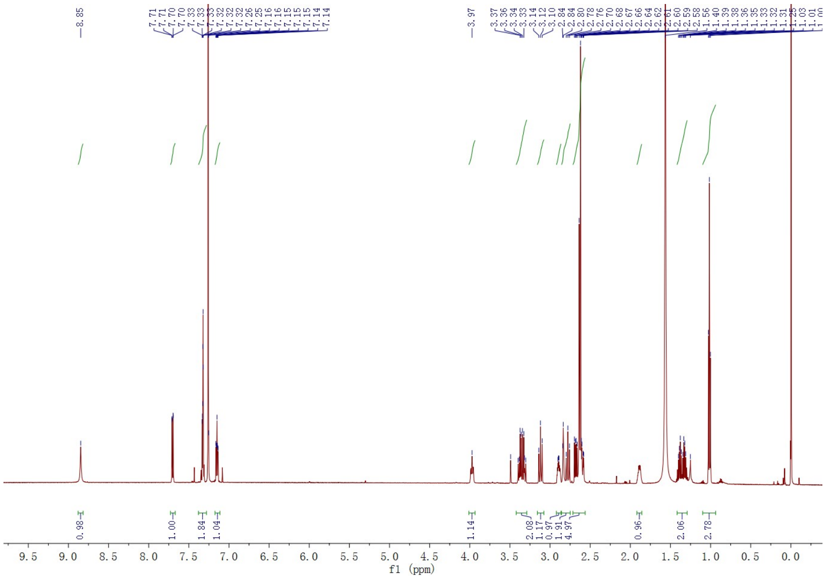

Figure S6. ^{13}C NMR spectrum of compound **2** in $CDCl_3$ (150 MHz)

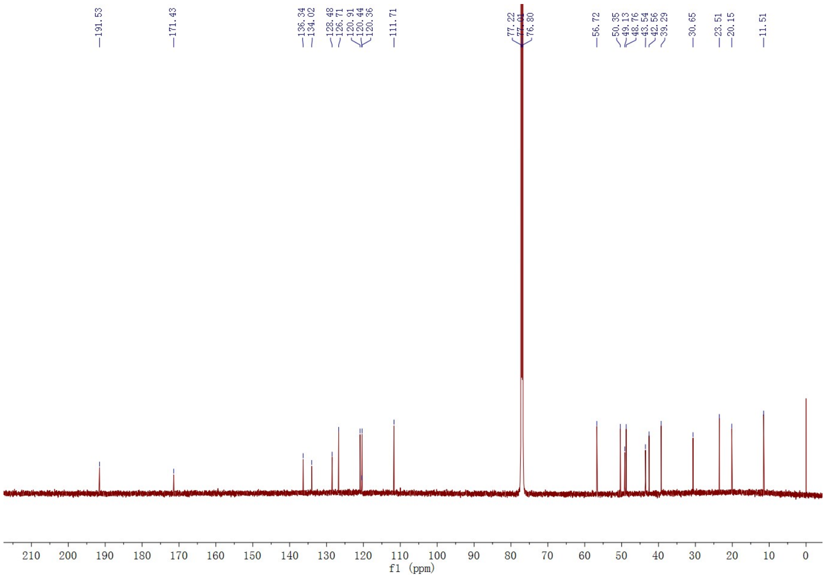

Figure S7. ESIMS of compound **3**

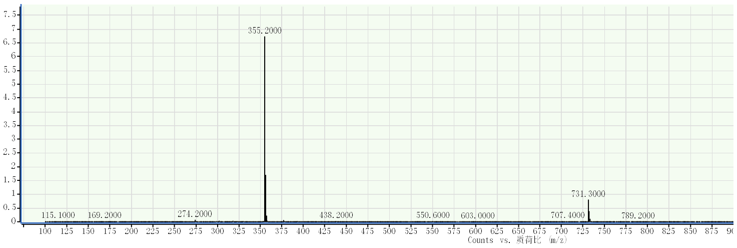

Figure S8. ^{1}H NMR spectrum of compound **3** in $CDCl_3$ (600 MHz)

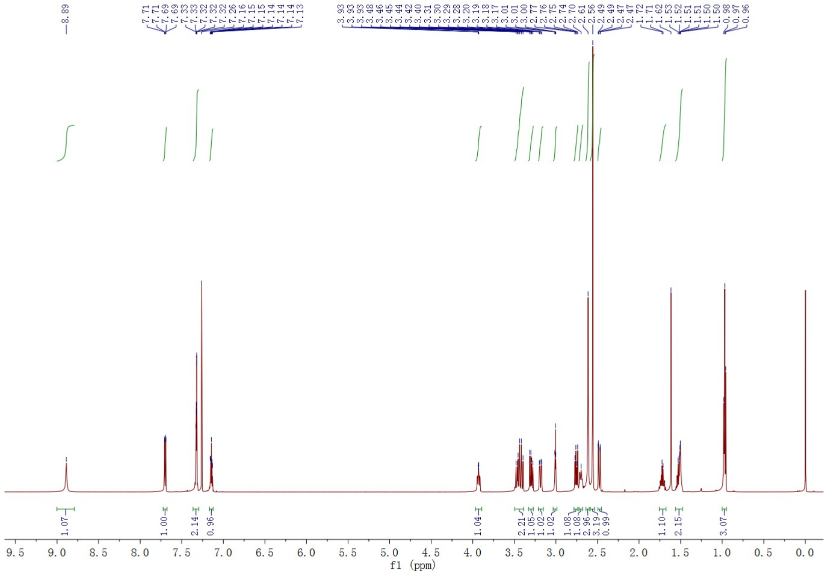

Figure S9. ^{13}C NMR spectrum of compound **3** in $CDCl_3$ (150 MHz)

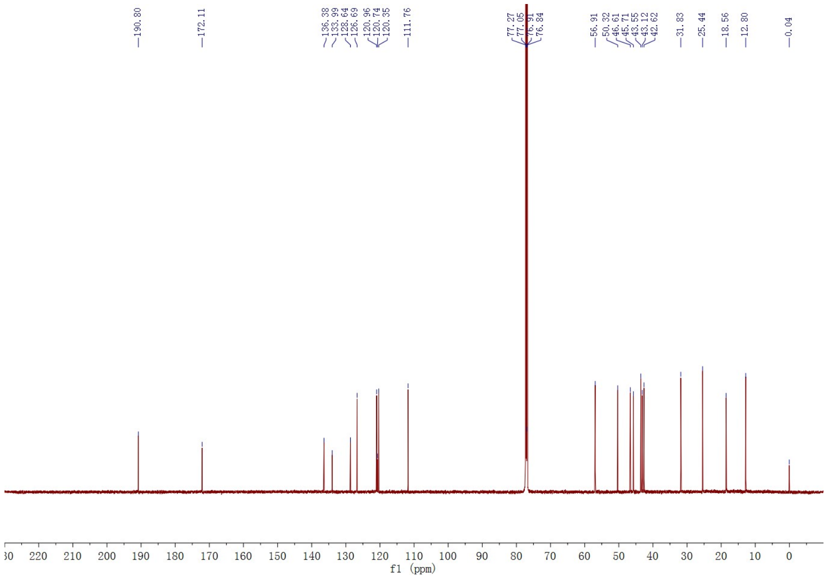

Figure S10. ESIMS of compound **4**

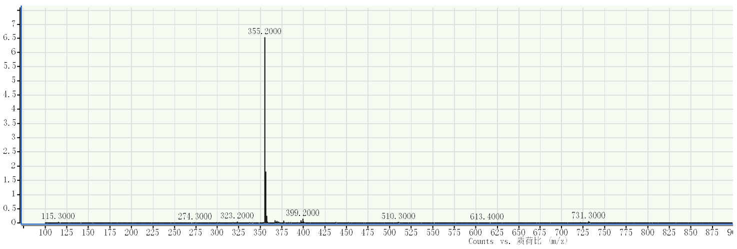

Figure S11. ^{1}H NMR spectrum of compound **4** in $CDCl_3$ (600 MHz)

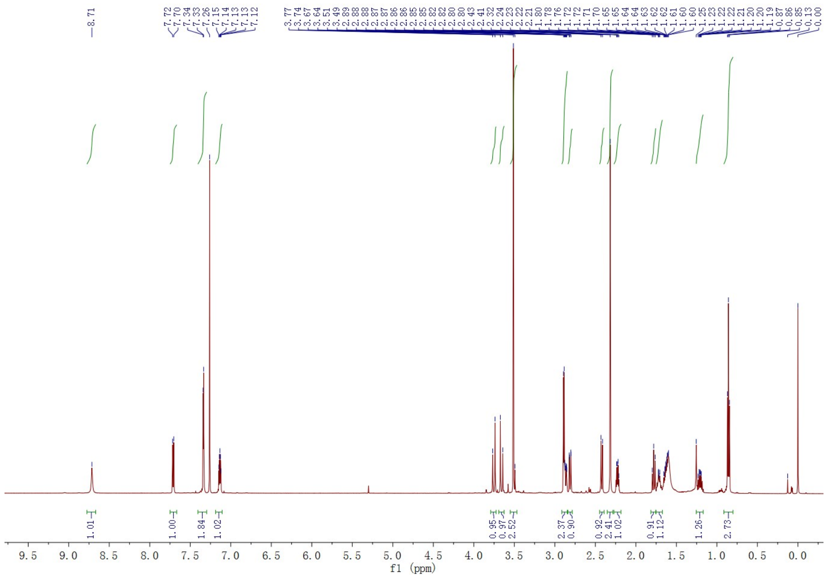

Figure S12. ^{13}C NMR spectrum of compound **4** in $CDCl_3$ (150 MHz)

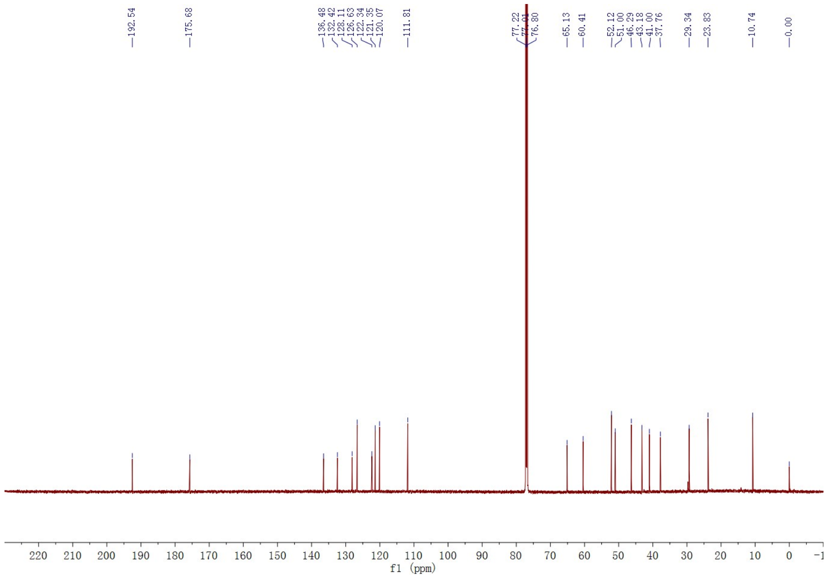

Figure S13. ESIMS of compound **5**

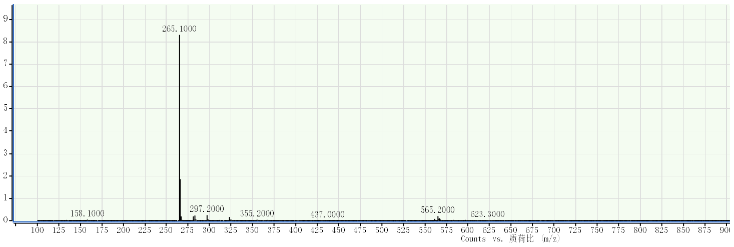

Figure S14. ^{1}H NMR spectrum of compound **5** in $CDCl_3$ (600 MHz)

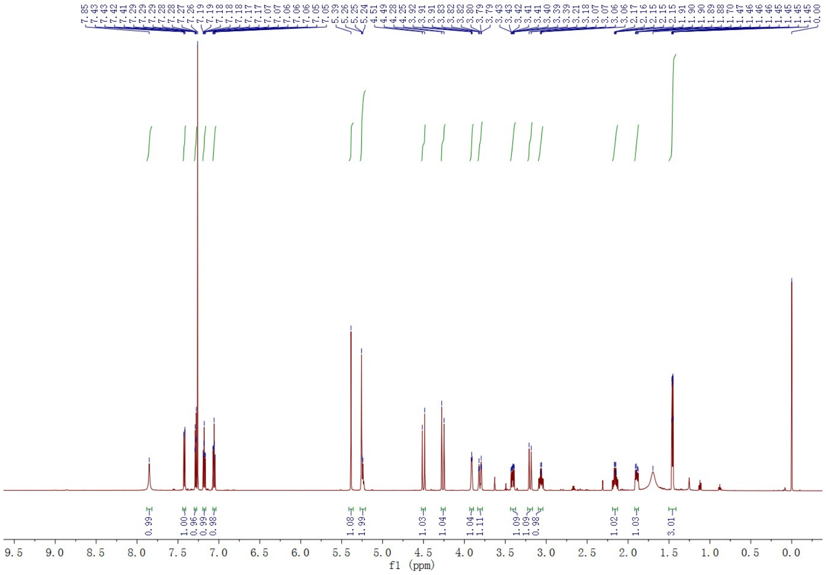

Figure S15. ^{13}C NMR spectrum of compound **5** in $CDCl_3$ (150 MHz)

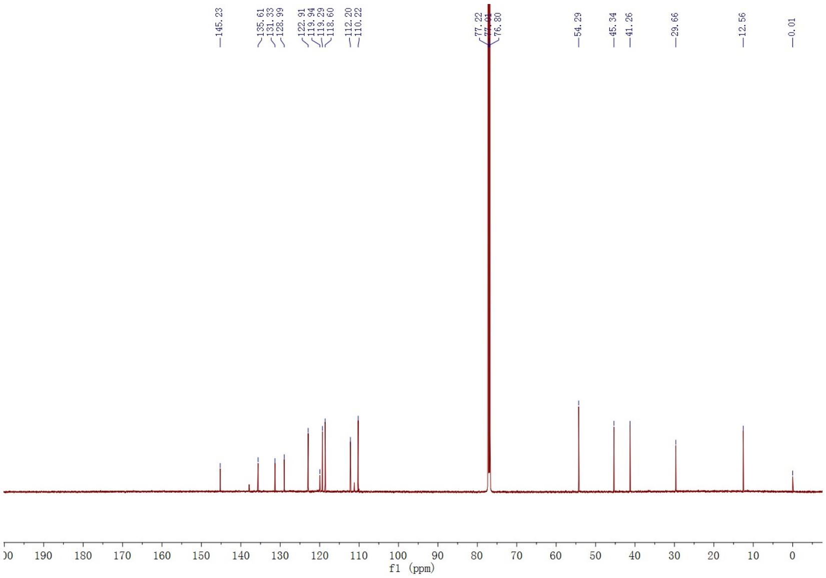

Figure S16. ESIMS of compound **6**

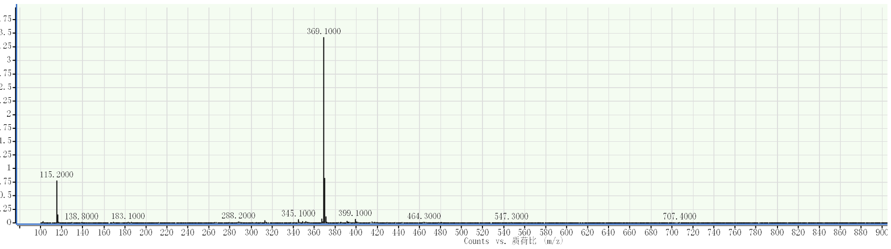

Figure S17. ^{1}H NMR spectrum of compound **6** in $CDCl_3$ (600 MHz)

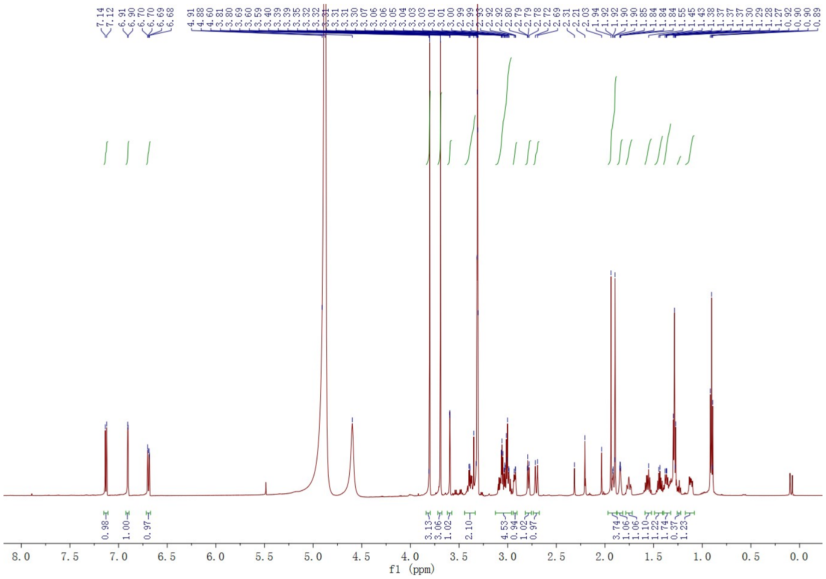

Figure S18. ^{13}C NMR spectrum of compound **6** in $CDCl_3$ (150 MHz)

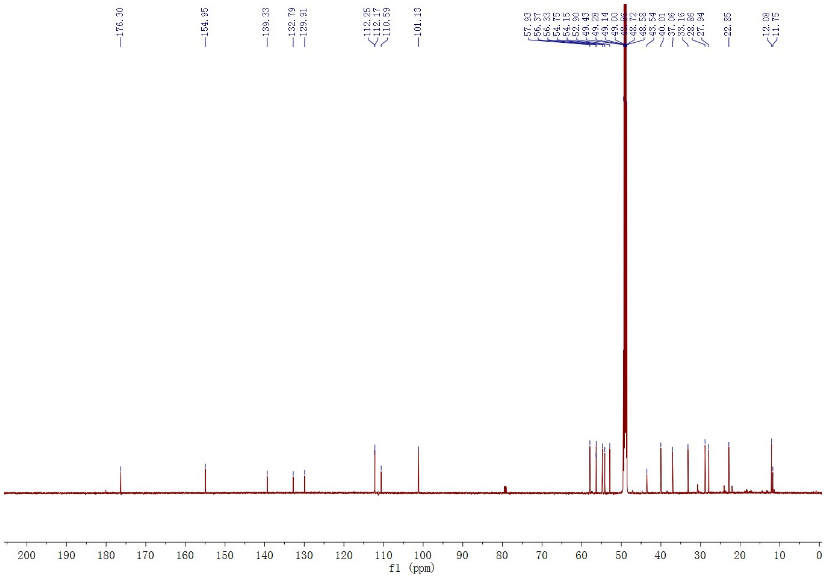

Supplement: Supplementary file 1 [file molecules-24-01316-s001.pdf]
